# Epidemiology and Control of *Plasmodium vivax* in Afghanistan

**DOI:** 10.4269/ajtmh.16-0172

**Published:** 2016-12-28

**Authors:** Toby Leslie, Sami Nahzat, Walid Sediqi

**Affiliations:** 1 London School of Hygiene and Tropical Medicine, London, United Kingdom.; 2 Health Protection and Research Organisation, Kabul, Afghanistan.; 3 National Malaria and Leishmaniasis Control Programme, Ministry of Public Health, Kabul, Afghanistan.

## Abstract

Around half of the population of Afghanistan resides in areas at risk of malaria transmission. Two species of malaria (*Plasmodium vivax* and *Plasmodium falciparum*) account for a high burden of disease—in 2011, there were more than 300,000 confirmed cases. Around 80–95% of malaria is *P. vivax*. Transmission is seasonal and focal, below 2,000 m in altitude, and in irrigated areas which allow breeding of anopheline mosquito vectors. Malaria risk is stratified to improve targeting of interventions. Sixty-three of 400 districts account for ∼85% of cases, and are the target of more intense control efforts. Pressure on the disease is maintained through case management, surveillance, and use of long-lasting insecticide-treated nets. *Plasmodium vivax* treatment is hampered by the inability to safely treat latent hypnozoites with primaquine because G6PD deficiency affects up to 10% of males in some ethnic groups. The risk of vivax malaria recurrence (which may be as a result of reinfection or relapse) is around 30–45% in groups not treated with primaquine but 3–20% in those given 14-day or 8-week courses of primaquine. Greater access to G6PD testing and radical treatment would reduce the number of incident cases, reduce the infectious reservoir in the population, and has the potential to reduce transmission as a result. Alongside the lack of G6PD testing, under-resourcing and poor security hamper the control of malaria. Recent gains in reducing the burden of disease are fragile and at risk of reversal if pressure on the disease is not maintained.

## Malaria Epidemiology and Risk in Afghanistan

Malaria occurs at altitudes below 2,000 m above sea level, and is most prevalent in snow-fed river valleys and areas used for growing rice. Transmission of *Plasmodium vivax* malaria takes place in May/June–November, with negligible transmission occurring between December and April. However, many *P. vivax* infections relapse during the spring season and this may give rise to a peak of clinical cases. The *Plasmodium falciparum* peak is shorter in August–October. The seasonality and relative low prevalence of malaria (which rarely exceeds 10% in the most endemic areas) results in a population only partly immune to malaria, with children and teenagers carrying most of the burden. *Plasmodium falciparum* is particularly unstable in this region, at the edge of its range, and can fluctuate markedly from year to year depending on climatic variation. Because of the infectious reservoir of hypnozoites and the ability of the disease to develop at lower temperatures in the vector, *P. vivax* affects a larger geographical area of the country than *P. falciparum*.

**Figure 1.**
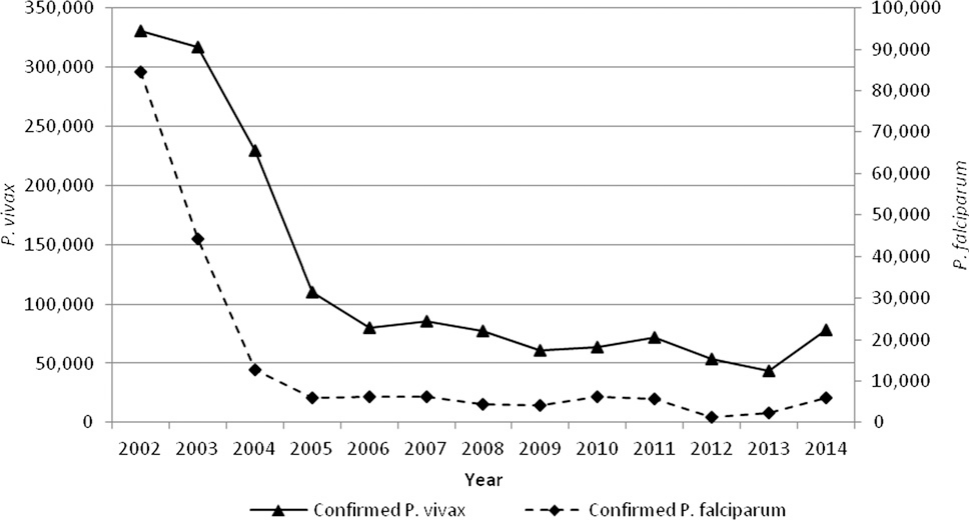
Number of confirmed cases of *Plasmodium vivax* (primary axis) and *Plasmodium falciparum* (secondary axis) in Afghanistan, 2002–2014.

Protracted wars from 1979 to 2002 had a significant influence on malaria transmission.^1^ The combination of an almost complete breakdown of health services and mass population movements resulted in an increase in malaria caseload from 40,000 to 80,000 per year in the 1970s to an estimated 2.5–3 million cases in 2002. This was compounded by the emergence of chloroquine-resistant *P. falciparum*, which changed the ratio of the two species to about 50:50 in some areas. With the introduction of effective treatments (first, sulfadoxine–pyrimethamine [SP], then artemisinin combination therapy [ACT] using SP and artesunate [AS]), the proportion of cases caused by *P. falciparum* has decreased from 13% in 2002 to 7% of confirmed cases in 2007 (6,283 cases), remaining at this level until 2014 (5,983 cases) (Figure 1

).

The health system has improved markedly and expanded, predominantly with funding from international donors. The number of basic health facilities in Afghanistan increased from 1,249 in 2004 to 2,015 facilities in 2010. The number of facilities able to diagnose malaria using a parasite-based test (mostly light microscopy) has also increased—more than 200 have been installed in clinics since 2004 in highly endemic areas. Normally, an improvement of case detection (i.e., from expanded services) would lead to an increase in reported cases as an artifact of improved surveillance. In Afghanistan's case, the consistency in the number of cases reported through the Health Management and Information System from 2004 to 2014 probably reflects an actual decrease in malaria incidence overall—numbers reported into the HMIS have remained constant, but the number of facilities reporting malaria cases into the surveillance system from endemic areas has increased.

Studies in the northern province of Kunduz in 2009, among basic health centers have shown slide positivity rate to be very low (< 0.1%) when slides are double read and confirmed by polymerase chain reaction (PCR) analysis.^2^,^3^ In the same study, by contrast, the eastern province of Nangarhar had a slide positivity rate of around 25%. Most of these cases were vivax malaria (98.7%, *N* = 446) versus six cases of falciparum malaria from June to September 2009.

In keeping with most settings, clinic microscopy in low-endemicity settings tends to overdiagnose malaria. The same studies in Kunduz and Nangarhar showed that as many as 80% of cases diagnosed in the clinics are false positives. Therefore, the burden of disease appears to be decreasing when viewed nationally.

Vivax malaria remains the major cause of malaria morbidity in Afghanistan. Reliable statistics regarding malaria mortality do not exist, although 32 deaths were reported in 2014. It is impossible to discern whether any deaths are attributable to vivax malaria because the reporting system does not differentiate the cause of death by species. It is assumed that any malaria death is attributable to falciparum malaria, but underreporting of vivax mortality is well documented.^4^

Afghanistan's malaria risk is heterogeneous and associated with environmental and human factors. The reduction in overall disease (e.g., in the North) has led to pockets of disease in foci. The improved surveillance and mapping of malaria has allowed assessment of the distribution of disease. In 2006, a risk-mapping exercise was undertaken to identify districts with high environmental risk of malaria.^5^

**Figure 2.**
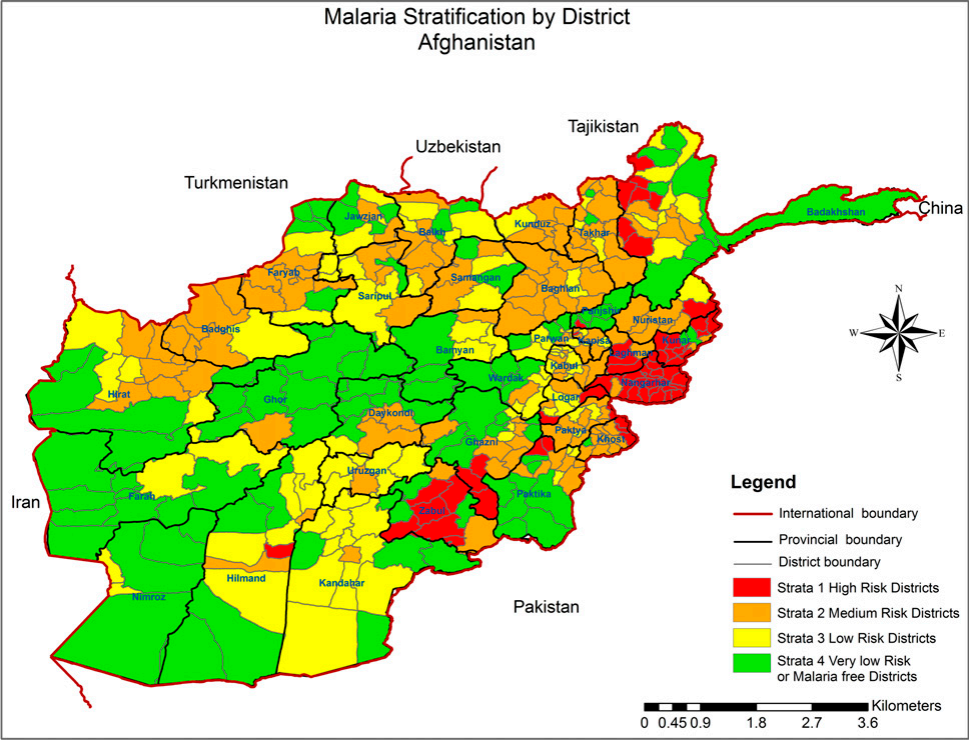
Malaria endemicity in Afghanistan at district level.

These areas are receptive to malaria transmission, but many of these areas currently have little active transmission. To improve targeting of malaria control resources, the risk-mapping data have been combined with recent routine reporting data on incidence and slide positivity rate to give risk models at district level (Figure 2

). For program delivery purposes, under the National Malaria Strategic Plan, the country has been divided into four risk strata: Stratum 1 districts (shown in red in Figure 2) with current ongoing high transmission, and incidence > 5 cases per 1,000 population per year. This comprises 63 of Afghanistan's 400 districts, contributing 84% of nationally reported malaria cases in 2013. Stratum 1 has a population of ∼3,000,000; Stratum 2 districts (incidence 1–5/1,000/year) with previous high transmission, which are receptive to transmission (138 districts); Stratum 3 districts with low transmission (incidence < 1/1,000/year) but with a risk of epidemics (96 districts); and Stratum 4 districts with no transmission but with clinical cases imported from other areas (103 districts), which are either too high or too arid and have never had active transmission. This more detailed malaria stratification underpins resource allocation for malaria control allowing a strategy of intensive focal control of disease at those areas with the highest burden.

## Malaria Vectors and Vector Control

Recent efforts to improve data on vectors, vector control, and insecticide resistance have enhanced knowledge of vector bionomics and vector control in Afghanistan.

An insecticide susceptibility survey conducted in 2010 highlighted the diverse distribution of primary and secondary vectors among several entomological sentinel sites in Afghanistan: *Anopheles superpictus* in Balkh (north) and Herat (western Afghanistan), *Anopheles pulcherrimus*, *Anopheles hycranus*, and *An. superpictus* in Kunduz and Badakshan (northeast), and *Anopheles stephensi*, *Anopheles subpictus* and *Anopheles culicifacies* in Nangarhar (eastern region).

**Figure 3.**
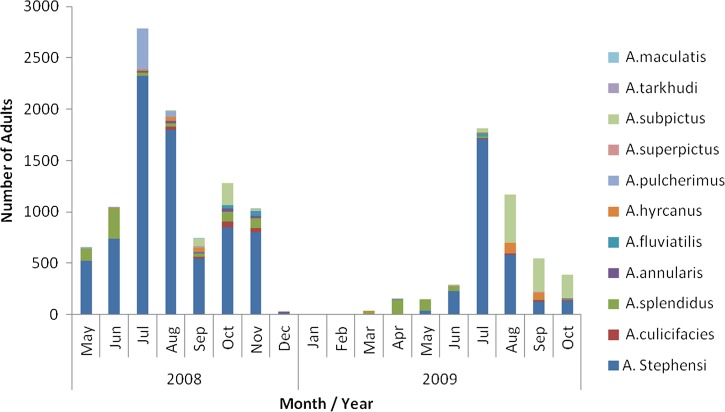
Relative abundance of *Anopheles* species in peri-urban and rural areas around Jalalabad city, Afghanistan, 2008–2010.

An extensive 18-month surveillance project from May 2008 to 2010 in and around Jalalabad city (Nangarhar Province, in the east of the country), in a high-transmission area of Stratum 1, showed the temporospatial distribution of mosquito fauna in four ecological zones (rice-growing agricultural land, non-rice-growing agricultural land, river margin, and peri-urban areas)^6^ (Figure 3

).

The study incriminates *An. stephensi* as the predominant vector, exhibiting strong zoophilic behavior^7^,^8^ and with a greater presence in animal sheds than in human habitations. *Anopheles stephensi* has been shown to be a primary vector of *P. vivax* (REF, Rowland).^7^ Abundance and species composition varied across the four ecological zones and included the following known vector species: *An. culicifacies*, *Anopheles fluviatilus*, *Anopheles annularis*, *An. pulcherrimus*, and *An. superpictus*. The relative role of these species in transmission of *P. vivax* and *P. falciparum* has not been fully studied in Afghanistan.

The country's first ever comprehensive assessment on the status of insecticide susceptibility among malaria vectors was conducted in 2010. The survey examined the susceptibility of wild caught *Anopheles* to three classes of insecticides (pyrethroids, carbamates, and organochlorines) in five provinces. The survey observed reduced susceptibility to all three classes of insecticides in Afghanistan, originating mainly in the eastern region (Nangarhar).^8^

Afghanistan has adopted integrated vector management (IVM) as part of the National Malaria Strategic Plan. This approach relies heavily on intersectorial collaboration to implement control measures based on factors which influence vector biology, transmission, and morbidity. An IVM steering committee was formed in 2010, which comprised members from key line Ministries such as Ministry of Agriculture, Irrigation and Livestock, Water and Power, Education, and Women's Affairs. However, little movement has been made toward practical steps for management of insecticide resistance.

Over the years, operational capacity building within the National Malaria and Leishmaniasis Control Programme (NMLCP) has improved through support of a range of donors to improve surveillance and undertake operational research projects. Efforts have now been harnessed to achieve “soft” and “hard” capacity development through the construction of insectaries, entomology laboratories, in Jalalabad, Kunduz, and in the future, Kabul. In addition to this, molecular diagnostics capacity building has been provided to give the first dedicated PCR and enzyme-linked immunosorbent assay laboratories in the NMLCP.

Capacity building of the entomology department continues to be an ongoing process to increase the abilities of the provincial program staff, enabling better surveillance of vectors, and insecticide resistance.

Long-lasting insecticide-treated nets (LLINs) are the mainstay of vector control in Afghanistan. In the last 4 years, more than 4,350,000 LLINs have been distributed with around 4 million distributed in high-burden districts of Stratum 1 provinces under this new strategy.

Afghanistan has adopted free house-to-house distribution of LLINs using a voucher system. Before the most recent risk classification (see above), this intervention was targeted at the population of 14 Stratum 1 provinces leading to an impressive increase in LLIN coverage rates at household and individual levels. A recent study conducted by the NMLCP^9^ described high coverage (> 60% households with at least one net in highly endemic areas), retention (> 90% of nets available 1 year after distribution), and high use of insecticide-treated nets (ITNs) in targeted areas (of those with a net, > 85% reported using them the night before). A recent study has evaluated the longevity of LLINs under operational conditions and shows that integrity and survival of LLINs was high after 4 years of use (unpublished report).^9^

Funding constraints have led to a change in strategy with universal coverage with LLINs being targeted at the 64 most highly endemic districts in the country. In other areas where the aim was previously for universal coverage, targeted distribution is being undertaken to prioritize pregnant women and children under 5 years of age. This is likely to lead to a decrease in population coverage in those areas in the coming years as coverage rates are subject to attrition as LLINs go beyond use.

LLINs are less effective at reducing the incidence of vivax malaria than that of falciparum malaria because relapse cases occur regardless of the use of an LLIN. This possibly accounts for the persistence of vivax as the main species when coverage of LLINs is high. A trial conducted in Pakistan in the 1990s showed a protective efficacy of ITNs of 62% against falciparum malaria versus 42% against vivax.^10^

## Diagnosis and Treatment of Malaria

Diagnosis and treatment of malaria is integrated into the Basic Package of Health Services in endemic areas in Afghanistan and so is provided free of charge. Microscopic diagnosis and rapid diagnostic tests (RDTs) are both in use, though clinical diagnosis without parasitological confirmation remains common at the level of the community health worker and in low-endemic strata. This approach leads to very high levels of overprescription of antimalarials with > 99% of malaria-negative fever patients being incorrectly treated with an antimalarial drug in very low endemic areas of northern Afghanistan.^2^ RDTs have been demonstrated to have advantages over both clinical diagnosis and microscopy,^11^,^12^ although both microscopy and RDTs suffer from low sensitivity and can lead to species misclassification.^13^ RDTs have also demonstrated utility when deployed at community level though the large network of community health workers in the country.

The biggest challenge to providing effective case-management services will be to maintain a sustained supply of tests which can reliably distinguish between *P. vivax* and *P. falciparum.* Because of resource constraints and a focus on malaria control (rather than local elimination), RDTs are being prioritized for use only in highly endemic districts. Recent data from 22 clinics in Afghanistan suggest that microscopy performance is suboptimal in both established laboratories and newly installed laboratories.^2^,^3^ Microscopists in both settings consistently overdiagnosed malaria (20–25% false-positive rate). There were also deficiencies in diagnosis of malaria in those with low parasite density (10% false-negative rate) and in detecting the rare cases of falciparum among many negative and a majority of vivax slides. The performance was also highly variable between clinics, indicating a need for constant training and support to quality assurance and control.

Afghanistan's treatment guidelines have been guided by local evidence from clinical trials and are based on World Health Organization–Eastern Mediterranean Regional Office guidelines. The clinical trials were prompted by insupportable levels of chloroquine resistance in *P. falciparum* malaria in the late 1980s^13^; in 1989, the failure rate of chloroquine- and amodiaquine-treated *P. falciparum* malaria has risen to more than 60% overall and was as high as 90% in Jalalabad.^14^ Trials continued into the use of the ACT, SP and AS 15,16 which was adopted for first-line treatment and also shown to be effective against vivax malaria.^17^

The status of SP resistance in falciparum malaria remains a concern, because the current combination with AS may mask any clinically observable resistance to SP. Molecular analysis of *P. falciparum* isolates has been conducted and will continue—a recent survey identified drug resistance alleles in increasing proportions.^18^ The combination remains effective after 10 years of use.^19^ Additional clinical trials may identify a different ACT (for example dihydroartemisinin–piperaquine), which could be used in all malaria cases regardless of species.^20^

For treatment of vivax malaria, trials have also demonstrated the continued efficacy of chloroquine to treat acute *P. vivax* cases. The efficacy of primaquine for radical cure of *P. vivax* is high, with PQ given over 14 days or 8 weeks preventing 60–90% of relapse cases,^21^,^22^ (Table 1 ). A trial also showed the poor efficacy of the 5-day regimen and led to its eventual abandonment across Asia.^23^ An often cited reason for the failure of primaquine is a lack of patient adherence to treatment, but a single trial in an Afghan population in Afghanistan showed that individuals with vivax malaria readily comply with a 14-day primaquine regimen if given appropriate instructions.^21^ However, despite the overwhelming evidence in favor of its efficacy, routine radical treatment using primaquine is almost never used because G6PD testing of malaria patients remains unavailable.

As in some vivax-endemic countries and in concordance with current WHO guidelines,^24^ the treatment guidelines provide a gold standard that is largely unattainable. In a case where there is a huge gap between policy and practice, the guidelines require treatment of vivax relapses with primaquine, stipulating that “The G6PD status of patients should be used to guide administration of primaquine for preventing relapse” but neither routine testing for G6PD deficiency nor primaquine is made available. Although the guideline is based on the several trials conducted in the region,^21^–^23^ the reality is that it has no realistic prospect of guidelines being adhered to unless G6PD deficiency can be readily diagnosed by treating clinicians.

*Plasmodium vivax* in Afghanistan has a long latency period before relapse. In one study, the time to the first recurrent episode was 66–74 days and there was no difference in the time period between those treated with 14-day primaquine and those who were untreated.^21^–^23^ This long latency period is what enables the subtropical strain of vivax to survive between transmission periods and may partially explain the longer lag-time to effective control.

The difference in incidence of malaria between those treated and those not treated with effective doses of primaquine (Table 1) indicates a protective efficacy of between 99% and 60% in those treated versus untreated with primaquine. The relapse risk over a long period of follow-up (9, 11, and 12 months in three trials) indicates that up to half of incident malaria cases may be due to relapse episodes. The main confounder that makes it impossible (at the present time) to clearly differentiate between relapses and new episodes or treatment failure is the inability to distinguish between new infections and relapses so it is not possible to define the burden of relapse with certainty. But in areas of very low transmission (such as the 2008 study in Table 1), there is a greater effect than in the earlier studies during times and in places of higher transmission. Thus, the impact of wide-scale use of primaquine would be to prevent a significant proportion of cases that result from relapses and to dramatically reduce transmission by reducing the infectious reservoir.

Adequate treatment of vivax malaria relapses is therefore now the main limitation in provision of effective treatment. If the gold standard treatment includes antirelapse therapy (primaquine), then almost none of Afghanistan's vivax malaria cases are adequately treated because of the absence of G6PD testing. G6PD deficiency is high in certain ethnic groups (up to 10% amongst male ethnic Pashtuns), and about 4% at the population level.^25^,^26^ The predominant G6PD genotype is the Mediterranean subtype, which is moderate to severe in the presence of hamolytic factors (e.g., primaquine, dapsone). This makes the risk of providing primaquine too high for widespread use without routine testing for G6PD deficiency, even though there is some preliminary evidence that G6PD deficiency is protective against infection with vivax malaria.^27^ The protective effect of G6PD is from a single case-control study and needs confirming in further studies. Even so, the effect is not absolute; so while it does potentially reduce the risk of hemolysis, it does not negate it completely. Little is known about the interaction of primaquine at therapeutic doses within females with heterozygous deficiency. Because heterozygous females produce a mixed population of deficient and nondeficient red cells (lyonization), it is unknown whether use of primaquine is safe in this group. Tests that can identify heterozygous females are not available at clinic level, but tests that can identify females with low G6PD deficiency could be used.

## Conclusions

Malaria control has reduced the incidence of disease over the last 15 years, with a rapid decline in *P. falciparum* malaria coinciding with an increase in the availability and use of ITNs and the introduction of more effective treatments. However, the same impact has not been seen in vivax malaria, which makes up the majority of malaria. *Plasmodium vivax* is currently the major challenge. Its control and elimination will be slower if conventional tools like LLIN and treatment of acute cases are not accompanied by an attack on the hypnozoite reservoir. Effective use of G6PD testing may allow improved access to primaquine, which would, in turn, reduce the ability of the parasite to maintain its infectious reservoir.

Reductions in available resources for malaria control or worsening of security could both have a major impact on the control and elimination of disease. Much of Afghanistan remains capable of supporting transmission (i.e., with the presence of vectors and humans) so reducing the pressure on the disease is likely to lead to resurgence. The widespread use of antirelapse therapy is likely to rapidly reduce the burden of disease, but this strategy relies on the availability of G6PD tests. In those who cannot be prescribed radical cure (pregnant and lactating women, infants, and those with G6PD deficiency), full protection through the use of ITNs is required.

## Figures and Tables

**Table 1:** Risk and odds ratios for relapse with vivax malaria from randomized control trials in Afghanistan and Pakistan (in Afghan refugees) on PQ treatment

Study	Period of observation (months)	Treatment arm*	Relapse risk	Odds ratio (95% confidence interval)†	*P* value
Leslie and others^22^	11	Placebo	22/71 (31.0%)	1	
	11	14-day PQ	1/55 (1.8%)	0.01 (0.002–0.1)	0.001
	11	8-week PQ	4/75 (5.1%)	0.05 (0.01–0.2)	0.001
Leslie and others^21^	9	Placebo	86/212 (40.6%)	1	
	9	14 day (supervised)	40/211 (19.0%)	0.35 (0.21–0.57)	0.01
	9	14-day (unsupervised)	34/173 (19.7%)	0.37 (0.23–0.59)	0.01
Rowland and others^23^	12	placebo	49/100 (49%)	1	
	12	14-day PQ	32/100 (32%)	0.6 (0.46–0.92)	0.014

PQ = primaquine.

* All groups received chloroquine.

† vs. placebo in the same trial.
